# Developing Solution-Processed Distributed Bragg Reflectors for Microcavity Polariton Applications

**DOI:** 10.1021/acs.jpcc.3c01457

**Published:** 2023-07-17

**Authors:** Emilia Palo, Michael A. Papachatzakis, Ahmed Abdelmagid, Hassan Qureshi, Manish Kumar, Mikko Salomäki, Konstantinos S. Daskalakis

**Affiliations:** † Department of Mechanical and Materials Engineering, University of Turku, FI-20014 Turku, Finland; ‡ Department of Chemistry, University of Turku, FI-20014 Turku, Finland

## Abstract

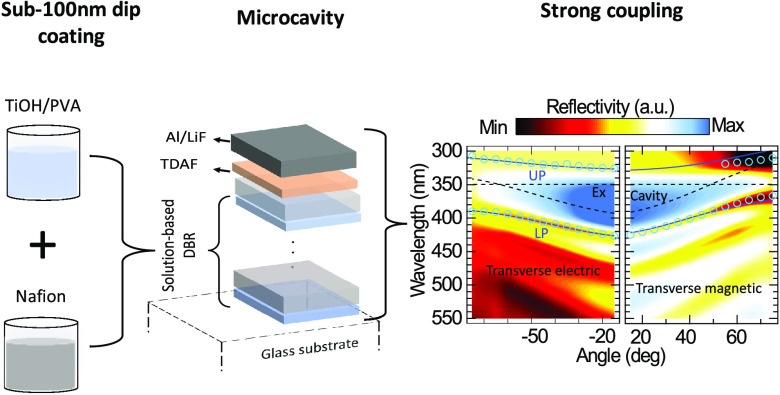

Improving the performance of organic optoelectronics has been under vigorous research for decades. Recently, polaritonics has been introduced as a technology that has the potential to improve the optical, electrical, and chemical properties of materials and devices. However, polaritons have been mainly studied in optical microcavities that are made by vacuum deposition processes, which are costly, unavailable to many, and incompatible with printed optoelectronics methods. Efforts toward the fabrication of polariton microcavities with solution-processed techniques have been utterly absent. Herein, we demonstrate for the first time strong light–matter coupling and polariton photoluminescence in an organic microcavity consisting of an aluminum mirror and a distributed Bragg reflector (DBR) made by sequential dip coating of titanium hydroxide/poly(vinyl alcohol) (TiOH/PVA) and Nafion films. To fabricate and develop the solution-processed DBRs and microcavities, we automatized a dip-coating device that allowed us to produce sub-100 nm films consistently over many dip-coating cycles. Owning to the solution-based nature of our DBRs, our results pave the way to the realization of polariton optoelectronic devices beyond physical deposition methods.

## 1 Introduction

Almost a century ago, Purcell^1^ paved the way for cavity quantum electrodynamics (CQED) by discovering that the spontaneous emission of an emitter is enhanced by modifying its electromagnetic environment. An optical microcavity is an electromagnetic resonator of light. It consists of two reflecting surfaces that are facing each other and are separated by a spacer layer with a thickness of *m*λ/2, where λ is the cavity mode light wavelength and *m* is the mode order. Highly reflective microcavity mirrors help to achieve a high quality factor (*Q*) optical mode, in which electromagnetic energy can be efficiently stored.^2^ In photonics, maximizing *Q* is highly desirable for both achieving large Purcell enhancement and facilitating the strong light–matter coupling regime.^3−6^ In this regime, the exciton resonance of the emitter and the cavity photon exchange energy faster than their individual loss rates. This leads to the creation of two new eigenstates called polaritons located above (upper polariton, UP) and below (lower polariton, LP) the exciton energy. The hybrid nature of polaritons, half-photon half-exciton, inherits them the ability to be delocalized over hundreds of nanometers while directly modifying the exciton energy levels. This unique potential of polaritonics to alter and often improve the optical, electrical, and chemical properties of materials has recently attracted much interest.^7^

Organic semiconductors emit light over the entire visible spectrum and can be deposited on any type and size of substrates inexpensively and with ease. All of the above have created a major trend in utilizing photonics for engineering the optical properties of organic semiconductors, particularly, in applications concerning organic light-emitting diodes (OLEDs) and lasers.^8−12^ Importantly, organic semiconductors are an excellent material set for achieving strong coupling because their exciton has large binding energy and oscillator strength. This has led to the exploitation of organic polaritonics in a plethora of optoelectronic research, such as photovoltaics,^13−20^ photodiodes,^21^ transistors,^22−24^ OLEDs,^21,25−33^ and lasers.^34−39^ In the case of polariton-emitting devices, such as OLEDs and lasers, a high-*Q* microcavity is important; this is needed for achieving sharp colors in OLEDs and low activation thresholds in lasers. While polaritons could improve the performance of solution-based optoelectronics, traditional methods for fabricating microcavities have been incompatible with solution-based methods.^40^

Metallic clad microcavities based on Ag, Au, and Al can be easily fabricated by inexpensive physical vapor deposition instruments, but they have only reached *Q* below 90.^41^ For polaritons to reach a high Q, the use of distributed Bragg reflectors (DBRs) is essential. A DBR is a periodic structure of thin layers of two or more materials with varying effective refractive indices. A stopband is the wavelength band of which the DBR is reflective due to constructive interference of radiation within the DBR. Dielectric DBRs have low absorption losses and a highly reflective and spectrally broad stopband. The latter is important for overlapping the cavity mode with the exciton resonance. In polariton microcavities, a DBR is made of two alternating dielectric materials, such as SiO_2_ and LiF as the low refractive index materials and SiN, TiO_2_, HfO_2_, Nb_2_O_5_, Ta_2_O_5_, and TeO_2_ as the high refractive index materials.^42,43^ These materials require sophisticated, resource- and time-demanding deposition processes, such as plasma-enhanced chemical vapor deposition (PECVD), physical vapor deposition, atomic layer deposition, molecular beam epitaxy and/or sputtering.^44^ They also often damage the layer onto which they are deposited, which is the case with sensitive organic layers. These bottlenecks can be overcome by fabricating DBRs with solution-processed methods, such as spin-coating,^45^ dip-coating,^46^ doctor-blading, and printing,^47,48^ which are advantageous over physical deposition methods in terms of processing, tuneability, scalability, and negative environmental impacts. However, there have been no efforts toward the realization of a solution-processed polariton microcavity.

**Figure 1:**
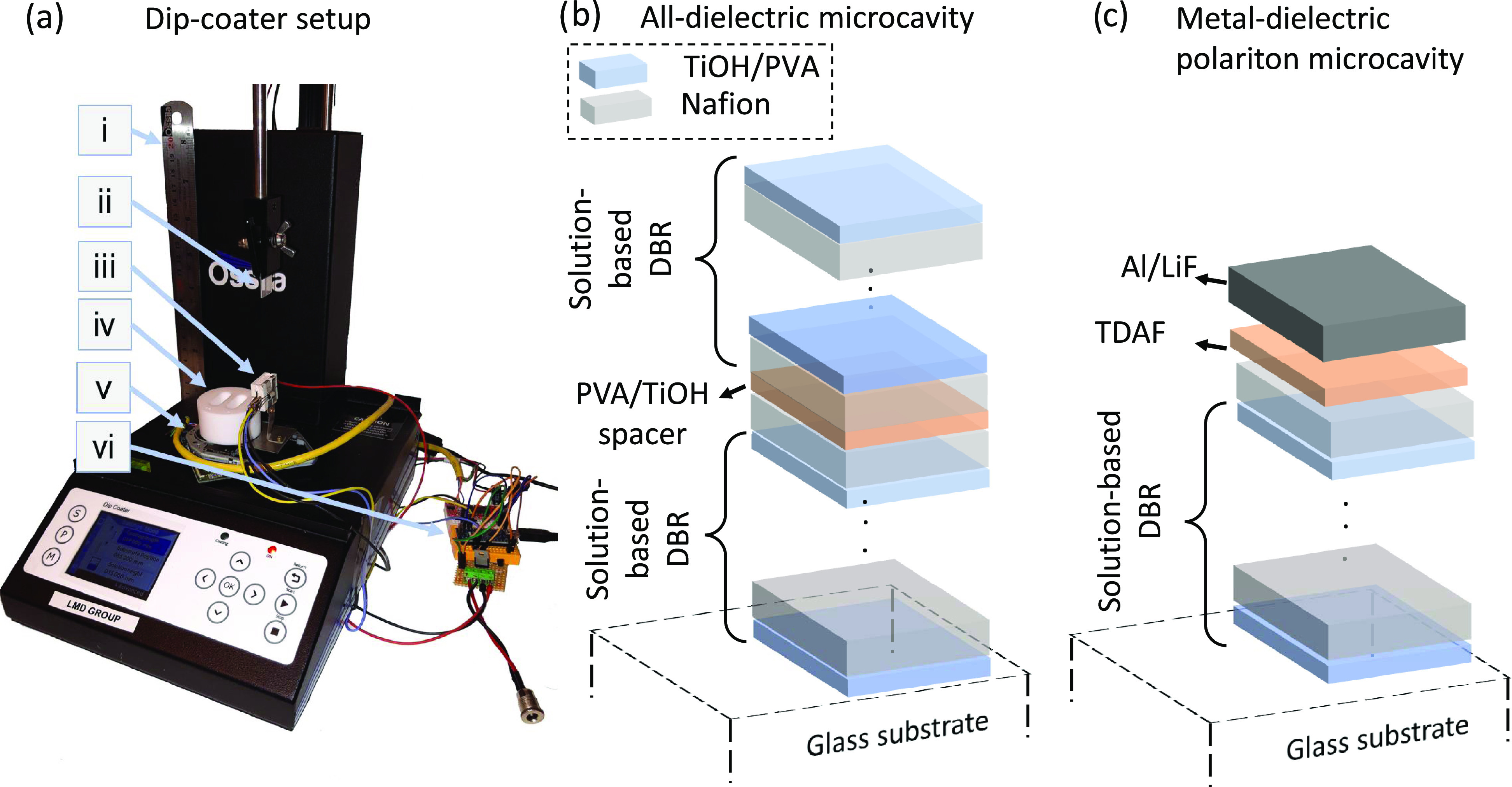
(a) Automatized dip-coating apparatus. (i) Ossila dip coater. (ii) Substrate (quartz and silicon were used). (iii) Heater for annealing the samples. (iv) Solution vessel that consists of two slots for PVA/TiOH and Nafion made from poly(tetrafluoroethylene). (v) Motorized linear stage that moves the vessel and the heater to alternate solutions and anneals the sample. (vi) Microcontroller-based control board that governs the whole automation process. (b) Schematic of an all-dielectric microcavity is shown that consists of two 6-pair DBRs with a bilayer of PVA spacer (around 140 nm thickness) in between. (c) Schematic of a hybrid metal–dielectric polariton microcavity is shown consisting of a bottom 6-pair DBR and a top 80 nm aluminum mirror and 5 nm LiF with 50 nm TDAF in between.

Here, we experimentally demonstrate, for the first time, strong coupling in an organic semiconductor microcavity made of an aluminum mirror and a solution-processed distributed Bragg reflector (DBR) (Figure 1). To accomplish this, we developed a simplified dip-coating protocol for sequential deposition of sub-100 nm Nafion and titanium hydroxide/poly(vinyl alcohol) (TiOH/PVA) films and by engineering a fully automatized dip coater for consistent dip-coating over many cycles.

While this fabrication technique is capable of producing any number of DBR pairs, we chose DBRs of 6 pairs to balance the trade-offs between fabrication duration, reflectivity, and film uniformity. To gain insight into the *Q* of our microcavities, we fabricated an all-solution-processed microcavity by sandwiching a 140 nm thick TiOH/PVA layer between 6-pair DBRs shown in Figure 1b. This structure reached a *Q* > 91, which is among the highest reported values in sub-10 pair solution-processed DBRs,^49,50^ which demonstrates the importance of producing nanothick films consistently over many dip-coating cycles. Strong coupling was achieved by coating a 6-pair solution-processed DBR with the well-established polaritonic organic semiconductor 2,7-bis[9,9-di (4-methylphenyl)-fluoren-2-yl]-9,9-di(4-methylphenyl)fluorene (TDAF), followed by 5 nm LiF and an 80 nm thin aluminum film,^51^ as depicted in Figure 1c. The hybrid metal–dielectric polariton microcavity featured a clear anticrossing between the UP and LP at the exciton resonance (see Figure 4). The polariton mode dispersion is in excellent agreement with a coupled harmonic oscillator model with standard TDAF fitting parameters.^36,51^ Our results indicate the tremendous potential of utilizing solution-processed DBRs for polariton studies and establishing the compatibility between polaritonics and large-scale optoelectronics fabrication methods.

## 2 Experimental Methods

### 2.1 Fabrication

The substrates (15 mm *15 mm *1 mm) were cleaned with water and soap (3% Decon90), acetone, and isopropanol solutions to remove any residue from the surfaces. The substrates were sonicated for 10 min on each step and dried with a N_2_ purge. We used silicon substrates to measure the optical constants of the films with ellipsometry and a quartz substrate to grow the DBR, microcavity, and polariton structures. For thin-layer preparation, Nafion (D-520 with dispersion 5% w/w in water and isopropanol) was diluted into a desired concentration with IPA. The 50% TiOH/PVA (Mowiol 18-88, *M*_w_ 130,000) hybrid material was synthesized following the protocol by Russo et al.^52^ by slowly hydrolyzing 2.2 mL of TiCl_4_ into 20 mL of cold H_2_O in an ice bath. This cold solution was then combined 1:1 in volume with cold aqueous PVA (e.g., 15 g/L) to create the TiOH/PVA hybrid solution described by Bachevillier et al.^46^ The solutions (for example, 3% Nafion in IPA and 50% TiOH in aq 15 g/L PVA solution) were added into a poly(tetrafluoroethylene) custom vessel shown in Figure 1a(iv). All solvents used here are technical grade. An in-house automated Ossila dip coater with a heating element for annealing was used to perform the multilayer deposition (Figure 1a). As illustrated in Figure S4, in each deposition step, the substrate was lowered into the desired solution, maintained for 10 s to wet the surface thoroughly, retracted at 40 mm/min speed, and then dried at 80 °C on the heating element for one and half a minute in a normal atmosphere. After dip-coating, DBRs were formed on both sides of the substrate. We removed the DBR facing the heater by simply scraping it out with a wet towel. The layer structure schematic of the fabricated samples is presented in Figure 1b. The DBR microcavity was made by depositing a DBR on the substrate, double dipping the sample in TiOH/PVA for creating the spacer (approx. 140 nm), and then developing the top DBR with that same dip-coating process. To achieve strong coupling, we replaced the TiOH/PVA spacer in the cavity with a 50 nm thick film of TDAF with exciton at 3.5 eV and the top DBR with a 5-nm-thick LiF/80 nm thick aluminum top mirror. TDAF, LiF, and Al were deposited at high vacuum (base pressure of >8 × 10^–6^ mbar, Angstrom Engineering physical vapor deposition system) with rates of 1, 0.2, and 0.5 Å/s for TDAF, LiF, and Al, respectively.

### 2.2 Characterization

We used a deuterium-halogen light source and an OceanOptics USB2000 spectrometer, coupled to a fiber to obtain transmission spectra of the materials and DBRs. The light coming out from the fiber was focused on the sample, after which the transmission was collected with a second fiber coupled to the spectrometer. Ellipsometric analysis using a J.A. Woollam VASE ellipsometer was used to acquire the thicknesses and refractive indexes of the materials. We utilized a Xe lamp with a spectral range of 250–2500 nm to obtain the spectra, and the data were analyzed by fitting a Cauchy model in the transparency region of the films; for further details, see Figures S5 and S6. All films were deposited on silicon substrates prior to the thickness calibration. The dispersion of the cavity and polariton modes was obtained with a VASE ellipsometer and a custom-built angle-resolved imaging setup that can measure reflectivity and photoluminescence. More information about this experimental setup can be found in our previous work.^53^ In brief, the collimated light from a halogen lamp illuminated the sample through an objective with 0.75 NA. The reflected light was then collected with the same objective and the backfocal plane image was focused onto the entrance slit of the spectrometer, which was coupled to a two-dimensional (2D) CCD camera (1340 × 400 pixels). The reflectivity dispersion was then resolved in wavelength vs angle. In photoluminescence configuration, the samples were excited using 250 fs pulses at 375 nm and 200 kHz repetition rate (Light Conversion Pharos, Orpheus, and Lyra). The transfer-matrix method (TMM) was used to simulate the reflectivity profile of DBRs, and a coupled harmonic oscillator model was used to fit the polariton modes and extract the Rabi splitting energy.

## 3 Results and Discussion

### 3.1 Developing Solution-Processed DBRs

**Figure 2:**
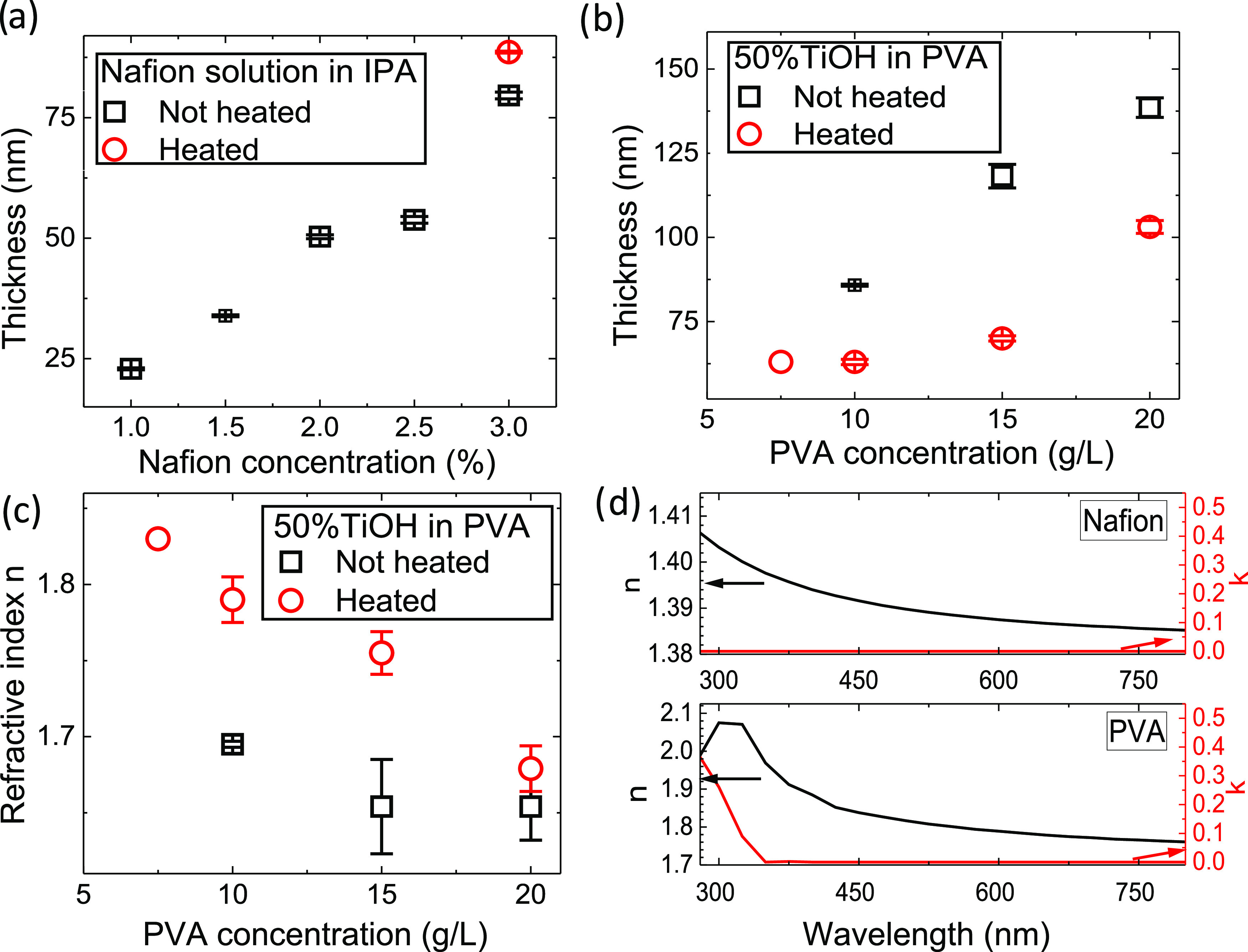
Properties of the single thin films of Nafion and TiOH/PVA. Thickness as a function of (a) Nafion and (b) TiOH/PVA concentrations. Note the thickness decrease of the TiOH/PVA film after annealing by ∼30 nm. (c) Refractive index of TiOH/PVA at 535 nm with and without annealing as a function of concentration. (d) Wavelength-dependent refractive index, *n*, and extinction coefficient, *k*, of 50%TiOH/PVA and 3% Nafion.

To fabricate DBRs that are highly reflective over a broad range of wavelengths, the two alternating layers should have a large refractive index contrast. In our case, we chose Nafion as the low refractive index material (1.35 at 500 nm) and titanium hydroxide embedded in the poly(vinyl alcohol) matrix (TiOH/PVA) as the high refractive index material (1.75 at 500 nm), resulting in a refractive index contrast of 0.4 at 500 nm. The refractive index of the TiOH/PVA layer depends on the thickness and amount of TiOH species in the layer but it is usually 1.7–2.0.^46,52^ Note that in this work, we used for the first time Nafion as the fluorinated polymer instead of (poly[4,5-difluoro-2,2-bis(trifluoromethyl)-1,3-dioxole-*co*-tetrafluoroethylene]) (PFP) used by Bachevillier et al.^46^ because it was readily available and inexpensive to acquire. The thickness and refractive index of the layers were tuned by changing the concentration of Nafion and TiOH/PVA (Figure 2a–d) while keeping the same withdrawal speed during deposition. The refractive index of Nafion was insensitive to its film thickness. In contrast, the annealing of TiOH/PVA resulted in a change in its film thickness and refractive index. Figure 2b,c shows that annealing the TiOH/PVA film at 90 °C decreased its thickness by ∼50% with a simultaneous increase of its refractive index by ∼6%. We attribute these changes to the evaporation of the interlocked water molecules in the TiOH/PVA film. For Nafion films, annealing decreased their thickness negligibly without affecting their refractive index. Because annealing provided a beneficial increase in the refractive index contrast between TiOH/PVA and Nafion layers, we decided to incorporate it into our DBR fabrication protocol.

In this work, we used 3% Nafion and 50% TiOH in PVA (aq) with a concentration between 15 and 7.5 g/L. The obtained refractive index profiles of the selected layers are shown in Figure 2d, and a detailed study of the refractive index with ellipsometry is shown in Figures S5 and S6. This fabrication flexibility is an essential element of our experimental approach that allowed us to easily modify the DBR design to match the respective TDAF exciton resonance at ∼3.5 eV for reaching strong coupling. The emission and absorption profiles of TDAF can be seen in Figure S2.

As shown in Figure 2d, both films exhibited negligible extinction coefficient (*k*) in visible with TiOH/PVA, showing increased *k* below 325 nm, which is attributed to TiO_2_ energy band gap. In this study, we considered two DBR architectures: one that was optimized for an all-solution-made dielectric microcavity with a stopband centered at 510 nm and the other with a stopband centered at 420 nm to accommodate a cavity mode near the exciton absorption of TDAF film at 355 nm, thus allowing us to demonstrate strong light–matter coupling using the exciton resonance for TDAF. It is worth noting that both DBR designs showed a broad stopband with FWHM >100 nm (>0.71 eV) and >160 nm (>0.78 eV) for DBRs centered at 420 and 510 nm, respectively, broad (Figure S1), which is attributed to the high refractive index contrast, 0.4, of Nafion and TiOH/PVA films.

### 3.2 Automation of Solution-Processed DBRs and Microcavities

**Figure 3:**
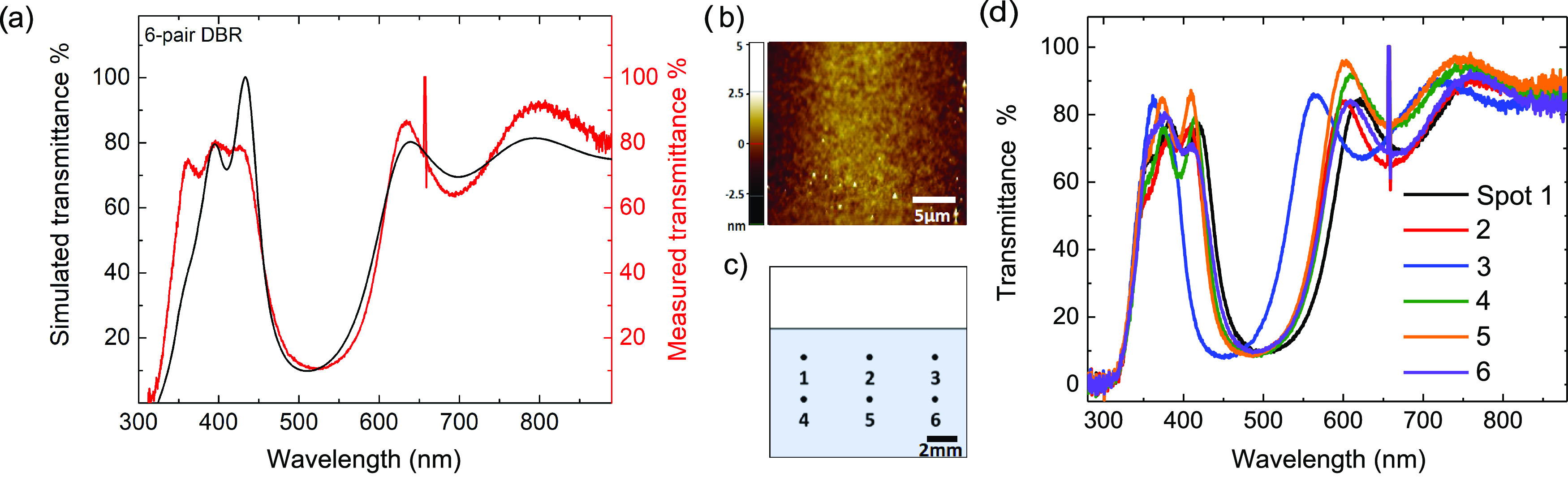
Transmission spectra of 6-pair DBRs. (a) Transfer-matrix (black) and measured (red) transmission are in excellent agreement. In the transfer-matrix simulation, we used the refractive indexes shown in Figure 2. (b) Atomic force microscopy image from a 25 μm^2^ scan of the DBR showing an RMS of below 1 nm. (c) Spatial mapping illustration depicting the measurement transmittance spots. (d) Transmittance spectra of spots in panel (c). Overlapping of transmission measurements in different spots clearly demonstrates an excellent uniformity, with the exception of spot 3 located at the edges of the sample where uniformity is expected to be poor.

Reducing the fabrication time and improving the consistency of DBRs are extremely important when considering real-world applications. Therefore, we developed an automated dip-coating setup based on an Ossila dip coater coupled with an in-house-developed solution switcher with annealing capabilities shown in Figure 1a and Supporting Information. The solution switcher setup incorporates a single linear motorized stage carrying a vessel that holds the Nafion and TiOH/PVA solution and the heating element, a limit switch which detects the retraction of the dip coater arm, and a microcontroller (Arduino Nano) that coordinates the alternating cycles of sample coating and annealing (illustrated in Figure S4). To avoid damage to the temperature sensor of the automation system, we set the temperature at 80 °C instead of 90 °C, which did not affect the overall quality of the DBRs. We initially concentrated on optimizing the automated DBR fabrication for the architecture with its stopband centered at 510 nm. Figure 3a shows a clear and highly reflective stopband centered at 510 nm that is in excellent agreement with the intended DBR design. A small inconsistency of measured and simulated transmission could be related to the different surface properties of the subsequent base layer during consecutive depositions, namely, the switching between layers of Nafion and TiOH/PVA hybrid. The spike at 656 nm originates from the deuterium-halogen light source that we used to measure the transmittance. To investigate the roughness of the films and DBR, we performed atomic force microscopy, where we found an RMS roughness of below 1 nm, and the results are shown in Figures 3b and S7. A modified TMM method could be used if film roughness was significant.^54^ To evaluate the uniformity of the solution-coated DBRs, we spatially mapped six different locations on the sample, as depicted in Figure 3c, and measured their transmission, as illustrated in Figure 3d. To study the uniformity of different locations on the sample, we used a 50 mm focal lens to focus the light from a deuterium-halogen source down to sub-500 μm spot. It can be clearly seen that the sample shows an excellent spectral uniformity throughout the coated area. In addition, the DBR optical response remained unchanged after storing it for four months in air, which indicates that the samples possess a long shelf life (Figure S1).

**Figure 4:**
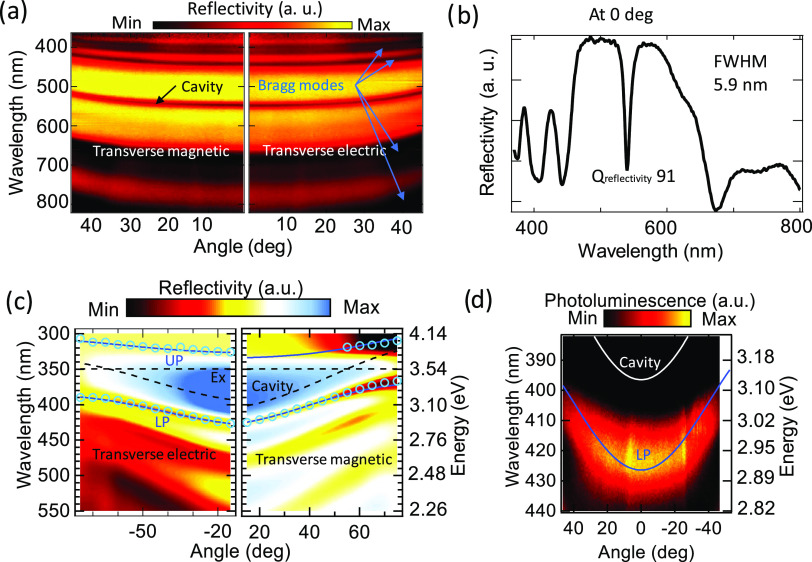
(a, b) Reflectivity of an all-solution-made dielectric DBR microcavity consisting of 6-pair DBRs and a 140 nm thick TiOH/PVA spacer layer. (a) Angle-resolved images for transverse-magnetic (left) and transverse-electric (right) polarization. A clear cavity mode can be seen at 521 nm (cavity, black arrow) and DBR Bragg modes (Bragg, blue arrows) with characteristic parabolic dispersion. (b) Reflectivity at a normal collection angle was used to calculate the *Q* factor. A sharp dip with a 5.5 nm full width at half-maximum results in a *Q* > 91. Note that this *Q* was achieved by using DBRs with only 6 pairs. (c, d) Reflectivity and photoluminescence were measured for 50 nm thick TDAF metal-DBR polariton microcavity. (c) Measured angle-resolved reflectivity for transverse-magnetic (right) and transverse-electric (left) polarization. The blue circles are the individual polariton minima from reflectivity spectra, shown in Figure S8, used to fit a coupled harmonic oscillator model. The simulated dispersions of polariton are shown as blue solid lines, and the uncoupled exciton resonance and cavity mode are shown as black dashed lines. (d) Angle-resolved photoluminescence from the same sample using the k-space photoluminescence setup.

To evaluate the *Q* of our microcavities, we deposited a 140 nm thick TiOH/PVA layer immediately adjacent to the bottom DBR, followed by the deposition of the top DBR. We used TiOH/PVA as a spacer to enable the deposition of the entire structure without interrupting the automated procedure. Figures 4a and S9 illustrate the angular-resolved all-solution-processed DBR microcavity for vertical and horizontal polarization and Figure 4b illustrates the reflectivity at normal incidence. Here, we can clearly see the excellent quality of the cavity mode, which has a dip of sub-6 nm full width at half-maximum, concomitant with the Bragg modes outside the reflectivity stopband. A *Q* of more than 91 was reached in a dielectric DBR microcavity with only 6 pairs, making it a very attractive solution for polaritonic applications.

### 3.3 Polaritons in a Solution-Processed Bottom DBR Microcavity

Motivated by the high Q of the all-solution-processed dielectric microcavities, we extended our design to fabricate a microcavity operating in the strong coupling regime. By simply modifying the deposition protocol and reducing the concentration of PVA from 15 to 7.5 g/L, we centered the stopband of the DBR at 420 nm to accommodate the exciton of TDAF (see Figure S2), demonstrating the advantage of using an automated dip-coating approach. In this study, we chose TDAF as the exciton layer because it is a well-known organic semiconductor for polaritonics that has been studied extensively.^36,51^ This allowed us to directly identify strong coupling by fitting a coupled harmonic oscillator model with previously reported parameters. Moreover, to avoid damage to the TDAF organic layer, we fabricated a hybrid metal/solution-processed DBR microcavity by thermally evaporating the TDAF layer onto the DBR, followed by a 5 nm LiF buffer layer and an 80 nm Al top mirror (see Figure 1c). By performing angular-resolved reflectivity, we observed a clear anticrossing between the UP and LP, which also exhibits the characteristic bending near the exciton resonance at large angles for transverse-magnetic and -electric polarizations (shown in Figure 4c). To obtain the Rabi splitting, Ω, we examined the angle-resolved reflectivity spectra shown in Figure S8 to identify the positions of individual polariton minima, which then were used to fit a coupled harmonic oscillator model. The fit is in excellent agreement with measurements and previous reports of strong coupling in TDAF microcavities.^36,51^ For transverse-magnetic polarization, we obtained a Rabi splitting, Ω, of 803 meV using the parameters of cavity detuning to be −402 meV and *n*_eff_ of 1.9. For transverse-electric polarization, Ω was found to be 750 meV and *n*_eff_ of 1.6. We attribute this smaller Ω for transverse-electric polarization to the low-contrast UP reflectivity minima and to our simplified coupled harmonic oscillator model that does not account for the angle-dependent penetration depth of the top DBR. A second cavity with a smaller detuning of −320 meV is shown in Figure S8.

By optically pumping the samples nonresonantly at 60° with an absorbed pump fluence of 1 nJ/cm^2^, we observed strong photoluminescence from the LP dispersion (Figure 4d). The optical excitation power was kept very low to avoid damage to the aluminum bottom mirror and block bimolecular annihilation processes, namely, singlet–triplet and triplet–triplet annihilation.^36^ From the photoluminescence of LP at a normal angle, we extract the LP lifetime to be 24 fs. This further confirms that the excitonic material (TDAF) remained emissive despite being deposited onto a solution-processed DBR and emission occurs through the LP mode, thus further verifying strong coupling.

## 4 Conclusions

We have experimentally demonstrated strong light–matter coupling and polariton photoluminescence in a solution-processed DBR/aluminum microcavity made by dip-coating alternating layers of Nafion and TiOH/PVA. In addition, we fabricated an all-solution-processed dielectric DBR microcavity with a *Q* > 91 despite using only 6-pair DBRs. A highly-controlled fabrication of DBRs with sub-100 nm layers was achieved by our in-house engineered automated dip-coating switcher, which also allowed us to reduce the DBR fabrication time. In this work, we used for the first time Nafion as the low refractive index material, which is inexpensive compared with the previous reports of fluorinated polymers.^46^ Currently, the available options of solution-based high refractive index films are limited and rely on a combination of a polymeric matrix with inorganic (nano)particles.^55^ This can lead to scattering losses and absorption in wavelengths below 400 nm. To overcome this bottleneck, efforts should be directed to the fabrication of purely polymeric solutions with a high refractive index. Nonetheless, the ease of fabrication offered by solution-processed DBRs is a promising approach for advancing the field of organic polaritonics, where transitioning to simple and inexpensive fabrication methods is beneficial. Moreover, we believe that our work opens new avenues for infusing polaritonics into organic and printed optoelectronics to tune their properties and improve their performance.

## Supporting Information Available

Transmission spectra of a 6-pair DBR fresh and after 4 months; TDAF absorption and emission as a function of wavelength; schematic representation of the automation setup; illustration of the DBR fabrication process; description of the automatized dip-coating setup; ellipsometry and depolarization analysis of Nafion film on the Si substrate; ellipsometry and depolarization analysis of TiOH/PVA film on the Si substrate; atomic force microscopy measurements; angle-resolved reflectivity spectra of polariton microcavities; angle-resolved reflectivity spectra of the empty microcavity (PDF)
