# Supplementary material for: Developing Solution-Processed Distributed Bragg Reflectors for Microcavity Polariton Applications
Source: J Phys Chem C Nanomater Interfaces. 2023 Jul 17;127(29):14255–62. doi: 10.1021/acs.jpcc.3c01457 (PMC10388359; doi:10.1021/acs.jpcc.3c01457)
Supplement: Supplementary file 1 — jp3c01457_si_001.pdf [file jp3c01457_si_001.pdf]

Supporting Information

Developing Solution-processed Distributed Bragg Reflectors for Microcavity Polariton Applications

Emilia Palo, Michael A. Papachatzakis, Ahmed Abdelmagid, Hassan Qureshi, Manish Kumar, Mikko Salomäki and Konstantinos S. Daskalakis

Corresponding authors: konstantinos.daskalakis@utu.fi

Contents

Supplementary Figure S1. Transmission spectra of a 6-pair DBR fresh and after 4 months.

Supplementary Figure S2. TDAF absorption and emission as a function of wavelength.

Supplementary Figure S3. Schematic representation of the automation setup.

Supplementary Figure S4. Illustration of the DBR fabrication process.

Description of the automatized dip-coating setup.

Supplementary Figure S5. Ellipsometry and depolarization analysis of Nafion film on Si substrate.

Supplementary Figure S6. Ellipsometry and depolarization analysis of TiOH/PVA film on Si substrate.

Supplementary Figure S7. Atomic force microscopy measurements.

Supplementary Figure S8. Angle-resolved reflectivity spectra of polariton microcavities.

Supplementary Figure S9. Angle-resolved reflectivity spectra of the empty microcavity.

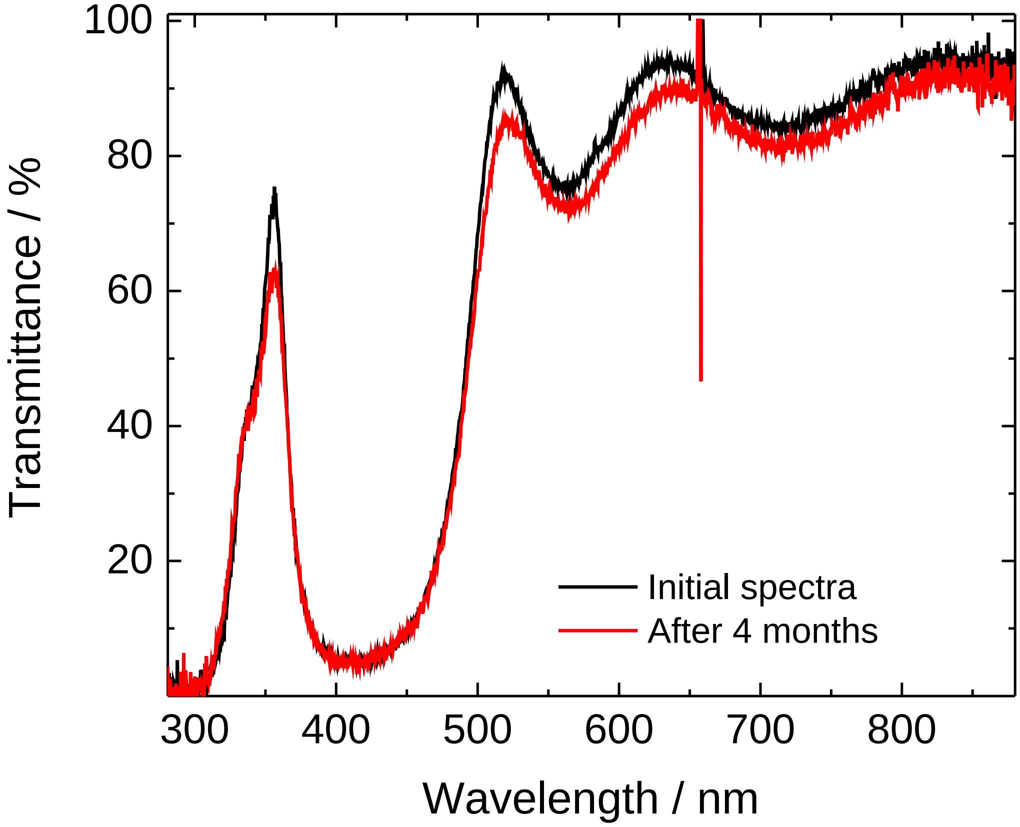

Figure S1: A comparison of transmission of a solution-processed DBR fresh and after four months. A slight decrease is observed at the Bragg modes but not in the stop-band region. We believe that this decrease is related to measured spot inconsistencies.

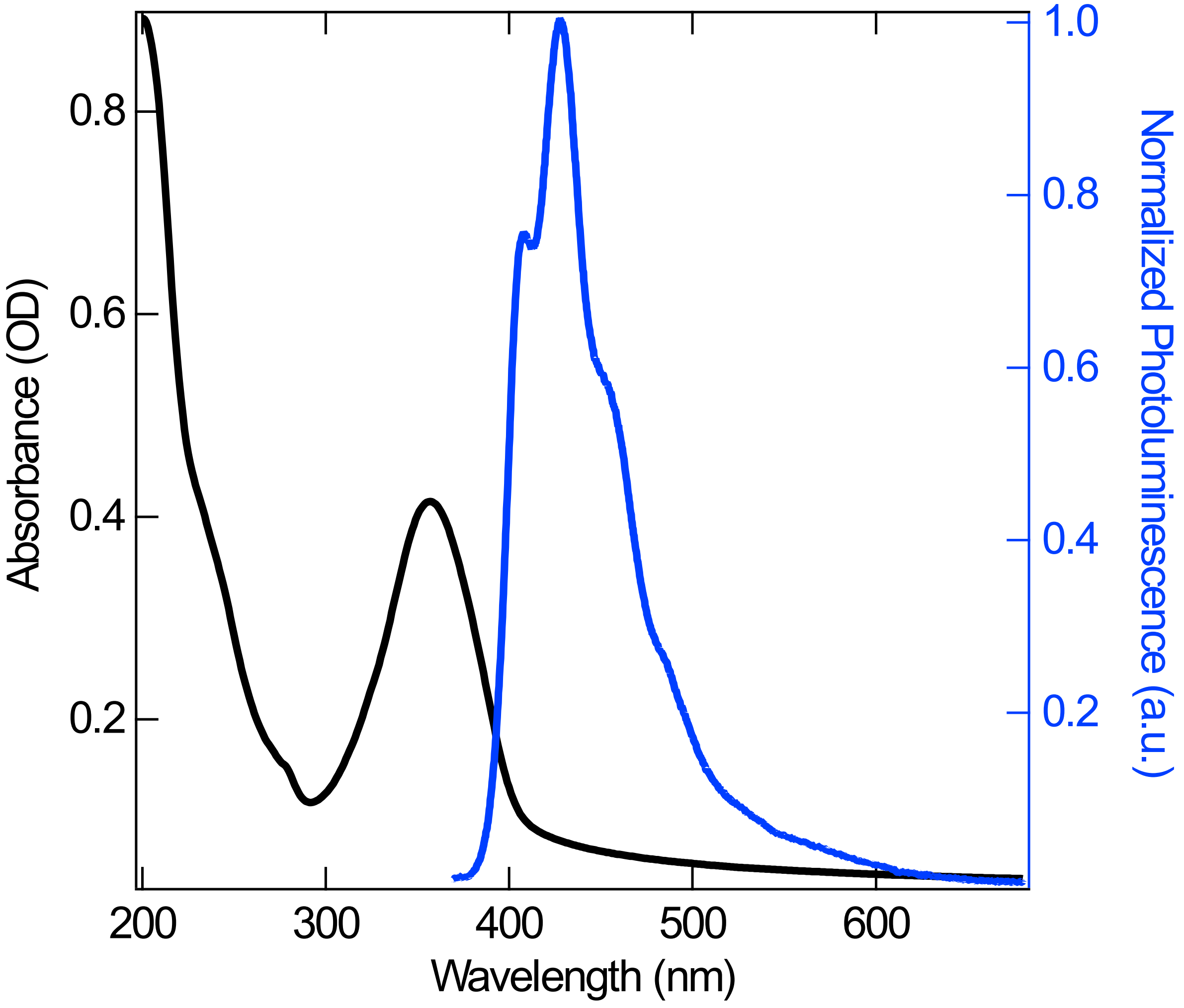

Figure S2: TDAF absorption and emission as a function of wavelength

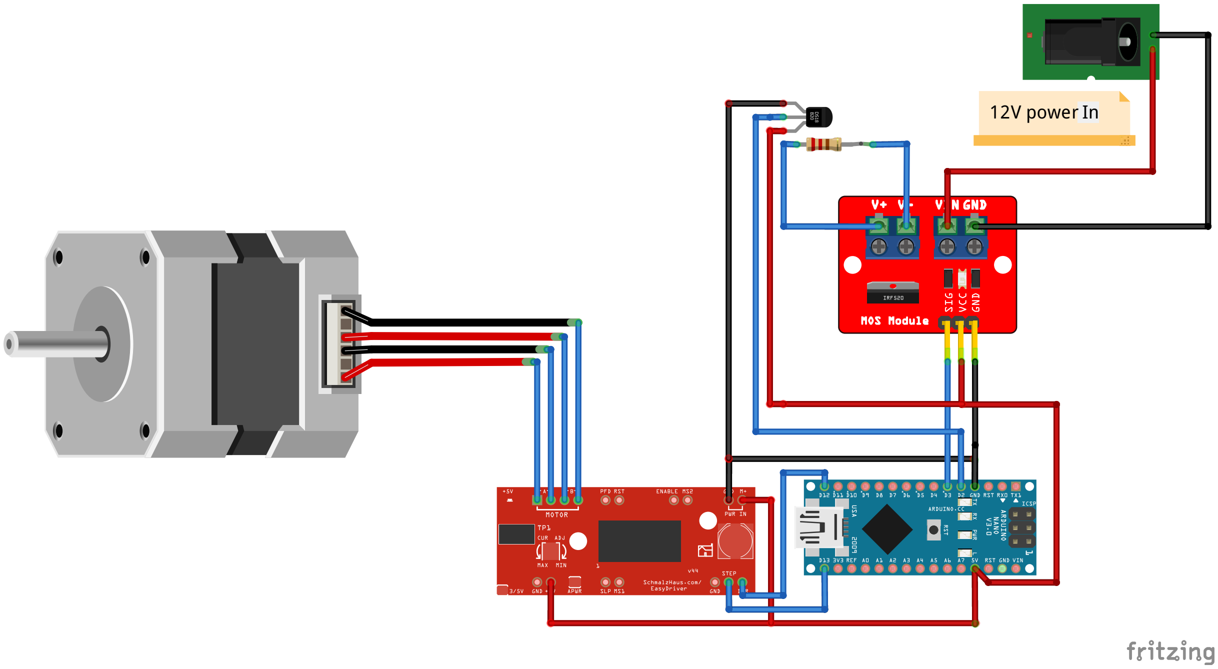

Figure S3: Schematic representation of the automation setup consisting of the linear stage's stepper motor, a stepper motor driver, a mosfet switch for the heater and finally the arduino nano development board.

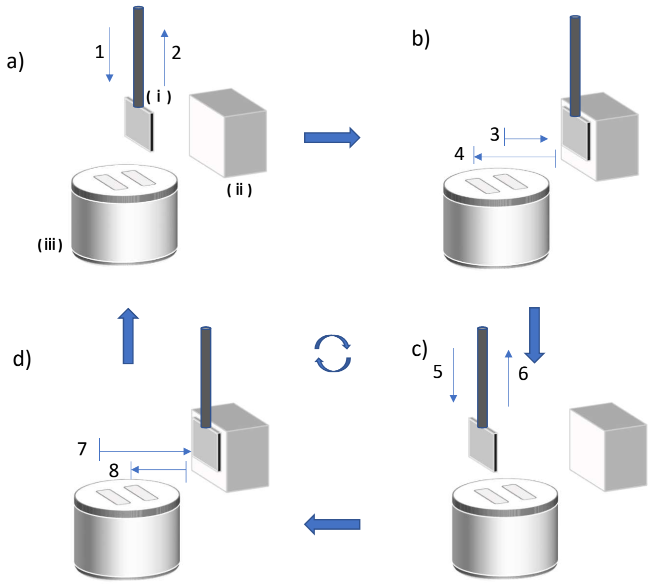

Figure S4: Illustration of the automatized dip-coating setup for the fabrication of DBRs and microcavities. The procedure is repeated for as many layers as its necessary. a) i) Dip-coater arm and substrate. ii) The heater block was used to anneal the samples. iii) Solution vessel that holds the high and low refractive index solutions. a) 1) The substrate is lowered into the first solution to start the first layer deposition. 2) The substrate is retracted from the first solution completing the first layer deposition. b) 3) The heater block approaches and comes in contact with the sample to anneal the first layer. 4) The substrate moves away from the heater block moving to the position of the second solution. c) 5) The sample is lowered into the second solution to start the second layer deposition. 6) The substrate is retracted from the second solution completing the second layer deposition. d) 7) The heater block approaches and comes in contact with the sample to anneal the second layer. 8) The substrate moves away from the heater block moving to the position of the second solution.

7 Description of the automated dip-coating setup.

The DBRs were made utilizing an in-house automatized Ossila dip coater using a solution switcher engineered from an old CD drive. This was done by utilizing the linear axis stage that was used to move the laser head (pictures in the supplementary). The automated switcher has only one degree of freedom yet it can switch the solutions and anneal the sample. The solution vessel was made from polytetrafluoroethylene and consists of two slots in which the solutions are placed. The substrate was lowered to the slot and then raised at 40mm/min to achieve the desired thickness. The motion of the linear axis was programmed using an Arduino nano development board and a stepper motor driver. A PID-controlled heater attachment on the linear stage consisting of two high-power resistors was used to anneal the sample and dry any solvents all in one movement without utilizing any complex mechanical structures. A switch was attached to the chassis of the dip coater to detect the movement of the dip coating arm. The stepper motor and the Arduino nano development board are powered with a 5V USB charger and the heater with a 12V brick adapter. The whole deposition of a pair of layers happens in only two stages. The setup has an extremely minimal cost (almost free from recycling old components) and this can be only accomplished because of the simplicity of dip-coating process. More information and code can be found here: `https://github.com/Michael-a-pap/dip_coater_mod`

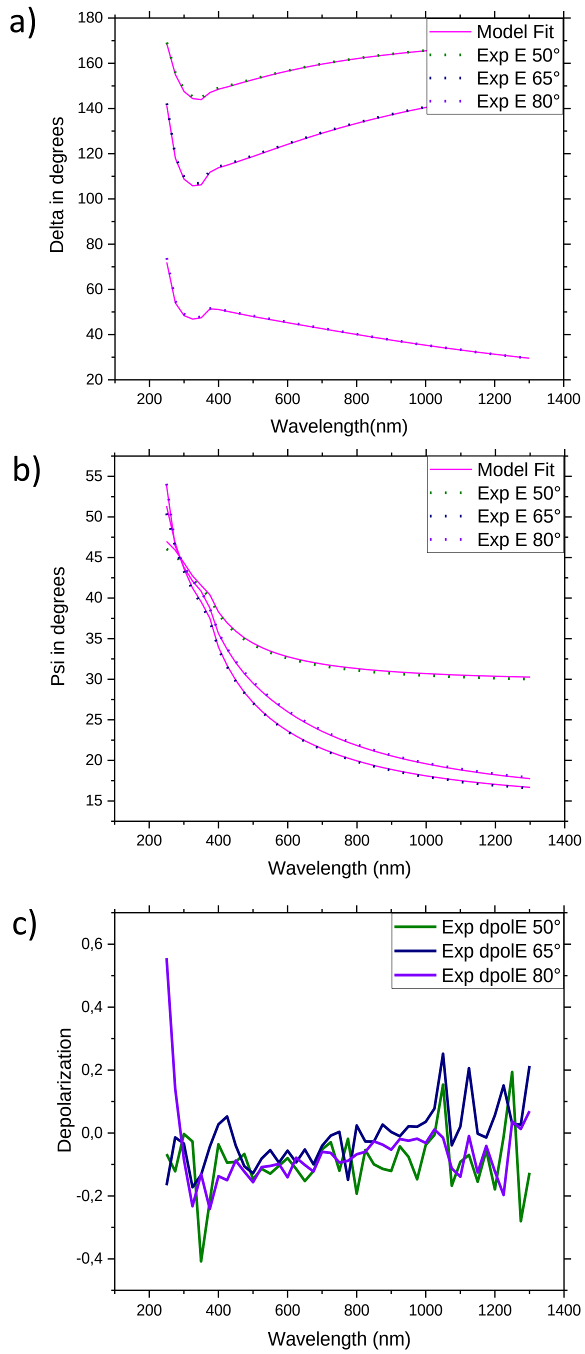

Figure S5: Generated and experimental for a) delta and, b) psi ellipsometric data for Nafion film on silicon, from the Cauchy fit with the film thickness fixed. c) Depolarization of Nafion film indicates negligible scattering.

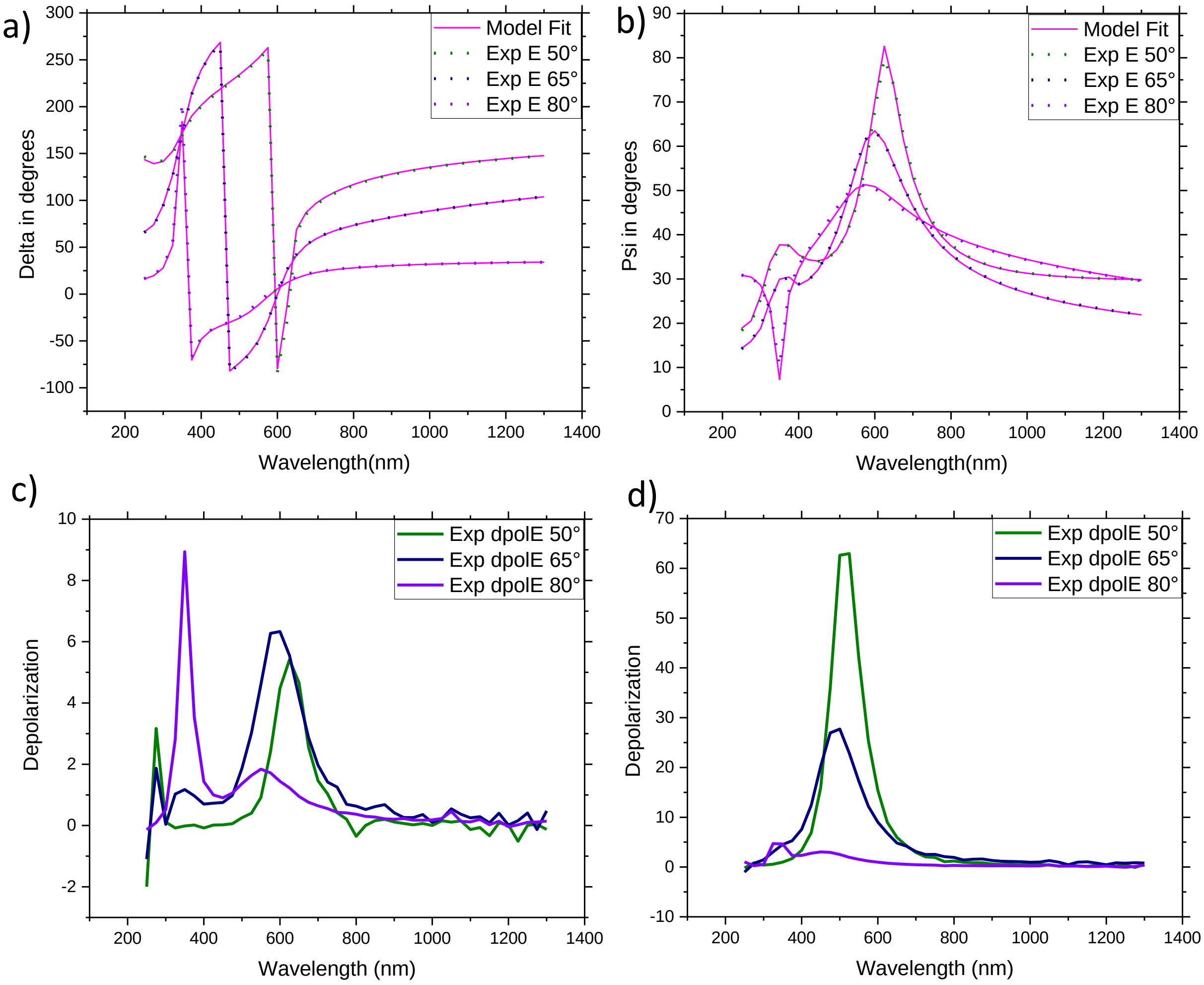

Figure S6: Generated and experimental for a) delta and, b) psi ellipsometric data for TiOH/PVA with 7.5 g/l PVA film on silicon. To accurately extract the refractive index we fitted Cauchy in the transparent region of the film (400-1300nm) and then performed a so-called "point-by-point" fit from 400 to 250 nm. This resulted in an excellent fit for both Delta and Psi data. Depolarization of TiOH/PVA film deposited from c) fresh and d) old solutions respectively. The depolarization of the film from the old solution shows significant scattering.

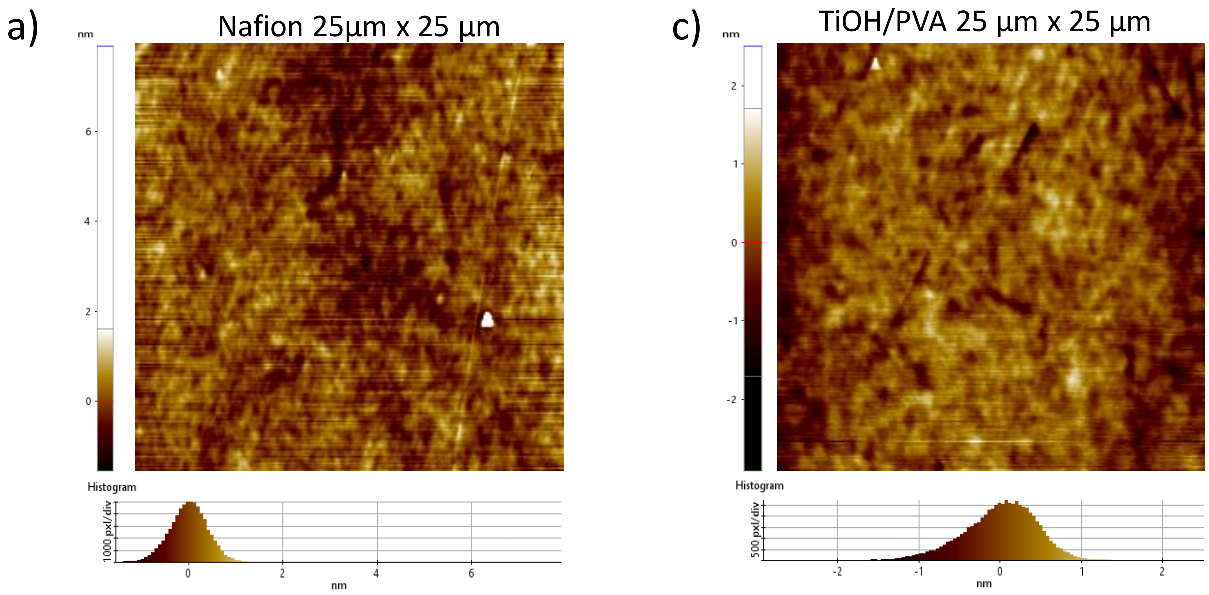

Figure S7: Atomic force microscopy measurements of solution-based films. Scans were performed over 25 μm 25 μm film surface areas of Nafion (a) and TiOH/PVA 7,5g/ml (b). All samples were found to exhibit RMS roughness of well below 1 nm.

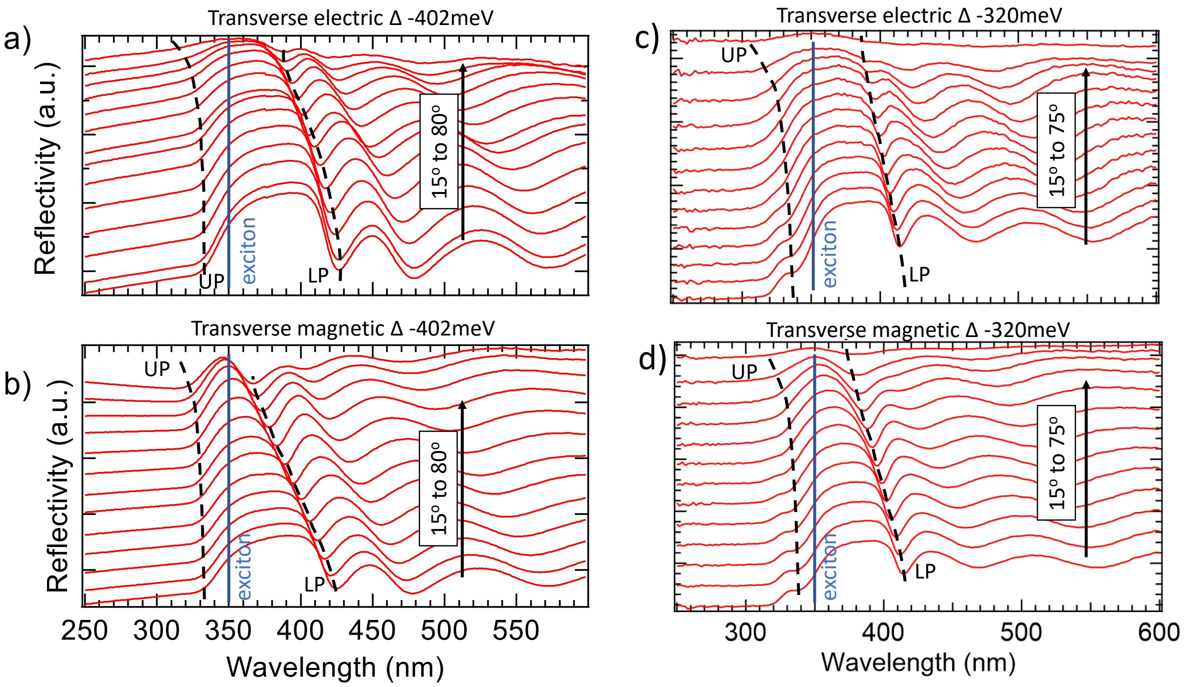

Figure S8: a), c) Transverse-electric and b), d)-magnetic angle-resolved reflectivity spectra of polaraton micorcavities shown for 2 different detuning of -402 meV and -320 meV. Polariton dips are difficult to identify from the contour plots of Fig.4 but can be seen clearly in the individual reflectivity spectra.

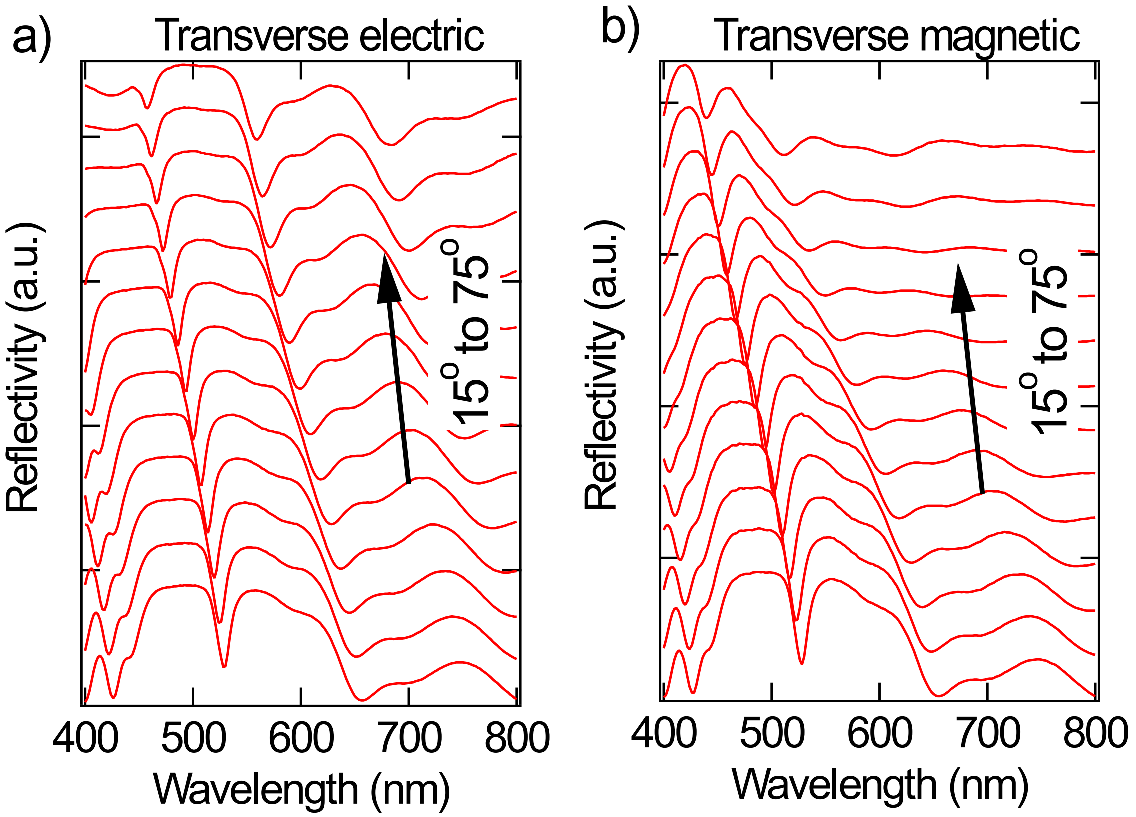

Figure S9: a) Transverse-electric and b)-magnetic angle-resolved reflectivity spectra of empty microcavity sample of Fig. 4a using the ellipsometer in reflectivity configuration to allow us to collect incidence angles beyond 45 degrees.
